# Acute and Sub-Acute Toxicological Evaluations of Bioactive Alkaloidal Extract from *Melodinus henryi* and Their Main Chemical Constituents

**DOI:** 10.1007/s13659-020-00252-2

**Published:** 2020-06-09

**Authors:** Meilian Yang, Yudan Wang, Zhifeng Fan, Qingwang Xue, Guy Sedar Singor Njateng, Yaping Liu, Jianxin Cao, Tianrui Zhao, Guiguang Cheng

**Affiliations:** 1 Faculty of Agriculture and Food, Kunming University of Science and Technology, Kunming, 650500 People’s Republic of China; 2 Engineering Research Center of Biopolymer Functional Materials of Yunnan, Yunnan Minzu University, Kunming, 650500 People’s Republic of China; 3 Department of Chemistry, Liaocheng University, Liaocheng, 252059 Shandong China; 4 State Key Laboratory of Phytochemistry and Plant Resources in West China, Kunming Institute of Botany, Chinese Academy of Sciences, Kunming, 650204 People’s Republic of China

**Keywords:** *Melodinus henryi*, Acute toxicity, Subacute toxicity, Monoterpenoid indole alkaloids

## Abstract

**Abstract:**

*Melodinus henryi* is a good source of terpenoid indole alkaloids, and traditionally used as a folk medicine in the treatment of meningitis and fracture. In order to further exploit their potential uses, its anti-inflammatory and immunosuppressive activities, safety evaluations and chemical profiles have been illustrated. Compared to the crude methanol extract from *M. henryi* and its non-alkaloidal fraction, the total alkaloidal fraction (MHTA) had the strongest anti-inflammatory and immunosuppressive activities. In the acute oral toxicity assay, the half lethal dose (LD_50_) of MHTA was more than 2000 mg/kg. The sub-acute toxicity assay for consecutive 28 days exhibited MHTA at a lower concentrations of less than 500 mg/kg might be regarded as safe, and might damage spleen, liver, kidney, and heart when the dose is higher than 1000 mg/kg. In addition, a phytochemical investigation on MHTA led to the isolation of 15 monoterpenoid indole alkaloids. Thus, in regard with the potent side effects of MHTA, it should be used with caution in the development of phytomedicine.

**Graphic Abstract:**

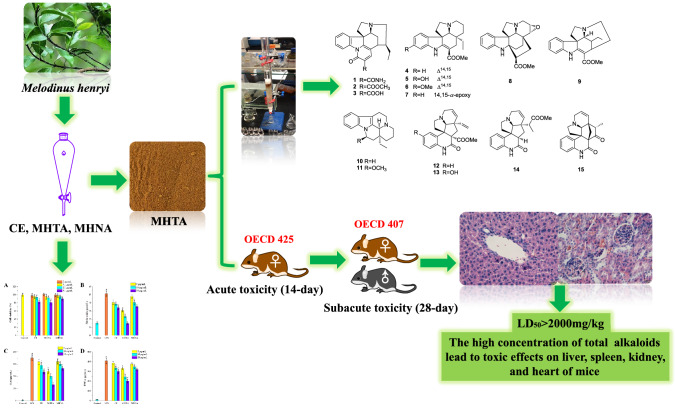

## Introduction

Medicinal plants have a long history in the prevention and treatment of human various diseases due to their health promoting potentials [1]. The natural products from medicinal plants are an important productive drug source for providing molecular scaffolds in the modern drug discovery research [2, 3]. Alkaloid is one of the most important classes of plant natural products with a large range of therapeutic properties, including anti-depressant, immunosuppressive, anti-microbial, anti-inflammatory, anti-tumor, etc. [4–6]. Some of which have been developed as clinical drugs to treat a variety of illnesses [7].

Plants of the genus *Melodinus* (Apocynaceae) are rich in terpenoid indole alkaloids (TIAs) and widely distributed in the tropical and sub-tropical regions [8]. Among them, *M. henryi* is a perennial vine mainly distributed in China, Thailand, and Burma [9] and has been used as a folk medicine for the treatment of infantile meningitis and fractures [9]. Phytochemical studies have revealed that the stems, bark, leaves, and fruits of *M. henryi* present a series of structurally novel TIAs in the meric and dimeric forms, such as melodinines A-G [10] and V [11], melodinines X1‒X4 [12] and W1-W4 [13], melohenines A and B [14], melodinhenines A-F, henrycinols A and B [15, 16]. Some of them were characterized with complex polycyclic skeletons and some alkaloids have significant biological potentials, especially the cytotoxic activity [10, 11, 17].

Previous studies of this plant are mainly focused on the discovery of TIAs with novel structures, and significant cytotoxic activity. However, the information of bioactive components responsible for the treatment of inflammatory diseases remains unclear. In order to further exploit its potential uses, it is necessary to illustrate the bioactive extract, evaluate its toxicity, investigate its chemical constituents. Thus, the obtained total alkaloidal extract (MHTA) and non-alkaloidal extract (MHNA) from *M. henryi* were evaluated their biological activities. Furthermore, the toxicological evaluations of MHTA were performed by assessing acute and sub-acute toxicities in vivo. The chemical constituents of MHTA were carried out by column chromatographic methods and identified by a combination with NMR and MS analysis.

## Results

### The Anti-Inflammatory Effects of Different Extracts from *M. henryi*

**Fig. 1:**
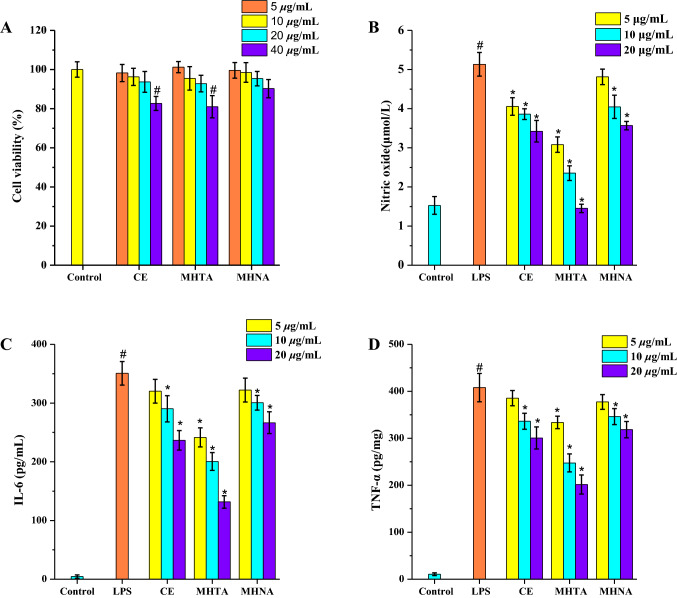
**a** Effects of CE, MHTA and MHNA on the viability of RAW264.7 cells; **b** effects of CE, MHTA and MHNA on nitric oxide production of RAW264.7 cells; The effects of CE, MHTA and MHNA on inflammatory cytokines of RAW264.7 cells, **c** IL-6; D: TNF-α. Data represent mean ± SD, *n* = 3. ^#^*p* < 0.05, means significant difference from the control group.*p < 0.05, means significant difference from the LPS group

The anti-inflammatory activity was performed in LPS-induced RAW264.7 macrophages as previously described method [18]. Firstly, the CE, MHTA and MHNA were evaluated their cytotoxic effects on the RAW264.7 cells. The cell viability results revealed that all the samples were of no toxic at the concentrations of 5–20 μg/mL (Fig. 1a). Thus, the anti-inflammatory effect of CE, MHTA and MHNA were investigated and the results were showed in Fig. 1b–d. As showed in Fig. 1b, the productions of NO increased by treating with LPS, and dose-dependently decreased by different extracts of *M. henryi*. Among them, the MHTA showed the highest inhibition of nitric oxide release (Fig. 1b). In addition, all the different extracts of *M. henryi* remarkably inhibited the levels of IL-6 and TNF-α compared to the LPS induced group. In particular, the MHTA showed the highest inhibitory effect on the products of pro-inflammatory cytokines.

### The Immunosuppressive Activity of Different Extracts from *M. henryi*

**Fig. 2:**
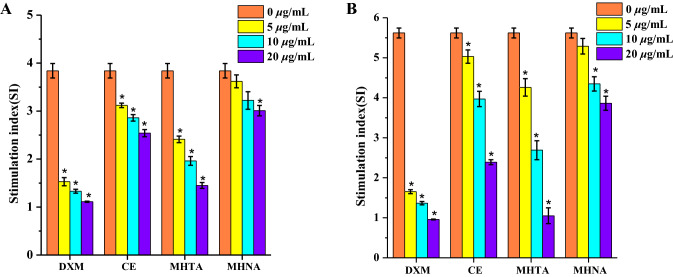
Inhibitory effects of CE, MHTA and MHNA on Con A-stimulated T lymphocyte proliferation (**a**) and LPS-stimulated B lymphocyte proliferation (**b**) in vitro. DXM was used as a positive control. The values are presented as mean ± SD of triplicates. *p < 0.05, means significant difference from the control group (0 μg/mL)

The immunosuppressive activity of CE, MHTA and MHNA were evaluated on mitogen-stimulated mice splenocyte proliferation with dexamethasone (DXM) as a positive control. As showed in Fig. 2a, b, different extracts of *M. henryi* significantly inhibited Con A-/LPS-stimulated mice splenocytes proliferation in a dose-dependent manner. Among them, MHTA showed the strongest inhibitory effect, comparable to the efficacy of DXM at a concentration of 20 μg/mL.

**Fig. 3:**
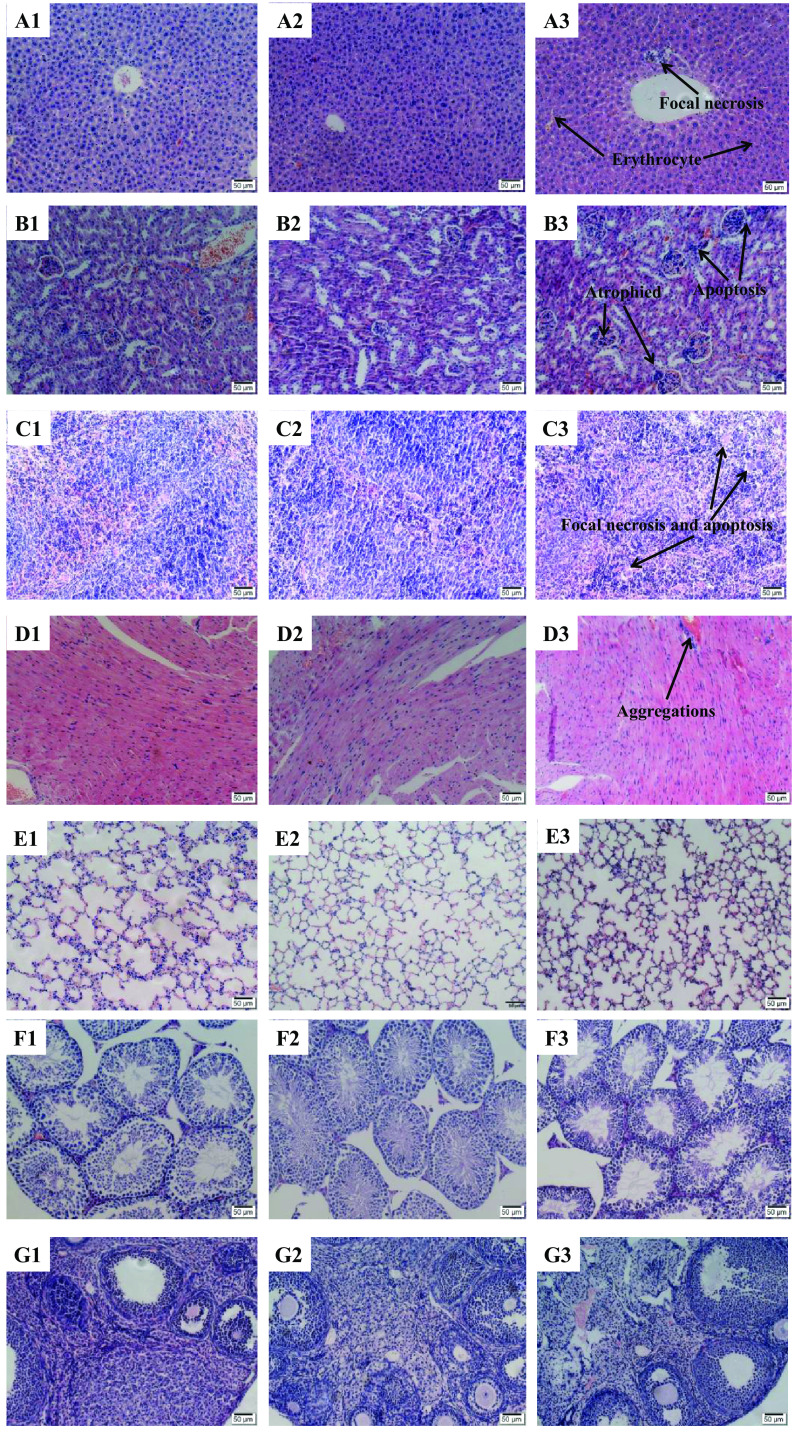
Histopathological results of seven organs in mice after oral administration for 28 days. Liver: A1 (Control); A2 (125 mg/kg of MHTA); A3 (1000 mg/kg of MHTA). Kidney: B1 (Control); B2 (125 mg/kg of MHTA); B3 (1000 mg/kg of MHTA). Spleen: C1 (Control); C2 (125 mg/kg of MHTA); C3 (1000 mg/kg of MHTA). Heart: D1 (Control); D2 (125 mg/kg of MHTA); D3 (1000 mg/kg of MHTA). Lung: E1 (Control); E2 (125 mg/kg of MHTA); E3 (1000 mg/kg of MHTA). Testis: F1 (Control); F2 (125 mg/kg of MHTA); F3 (1000 mg/kg of MHTA). Ovary: G1 (Control); G2 (125 mg/kg of MHTA); G3 (1000 mg/kg of MHTA). All tissues were stained with H&E (200 ×)

### Acute Toxicity Study

In the acute toxicity, oral administration of MHTA revealed no treatment-related mortality at a single dose (0, 300, 1000 and 2000 mg/kg). The food and water intakes and weight gain decreased slightly with no significant difference (*p* > 0.05) when compared to those of the control group (Table 1). The organ coefficients of body organs are shown in Table 2, and there were no significant variations among treated and control groups. Macroscopic examination of liver, kidneys, heart, and spleen did not reveal any changes. Therefore, the LD_50_ of MHTA was considered to be higher than 2000 mg/kg.

**Table 1:** Body weight gain, food and water consumption of mice treated orally with total alkaloid from*M. henryi*

	Acute toxicity (mg/kg)		Subacute toxicity (mg/kg)									
	Female		Female					Male				
	0	2000	0	125	250	500	1000	0	125	250	500	1000
Initial weight (g)	27.89 ± 0.56	27.12 ± 0.73	25.17 ± 0.86	24.99 ± 0.84	23.07 ± 1.28	26.67 ± 1.23	25.22 ± 0.88	32.04 ± 0.91	31.26 ± 0.81	31.26 ± 0.44	31.27 ± 0.89	32.65 ± 0.23
One week (g)	31.41 ± 1.64	30.98 ± 0.61	28.07 ± 0.75	26.50 ± 1.52	26.77 ± 0.99	27.70 ± 0.57	26.05 ± 0.14	34.81 ± 0.89	34.71 ± 0.91	35.29 ± 1.02	31.30 ± 1.31	32.95 ± 2.12
Two week (g)	33.49 ± 1.07	32.11 ± 0.78	30.01 ± 1.02	27.86 ± 1.02	27.70 ± 0.75	29.14 ± 0.63	27.92 ± 0.55	37.91 ± 1.32	35.83 ± 0.81	35.83 ± 0.76	33.62 ± 0.95	34.68 ± 1.42
Three week (g)	-	-	31.24 ± 0.78	29.68 ± 1.02	28.98 ± 0.55	31.37 ± 1.14	27.37 ± 0.99	39.58 ± 1.43	37.53 ± 1.77	37.32 ± 1.54	35.02 ± 1.47	36.38 ± 1.02*
Final weight (g)	-	-	33.97 ± 0.97	32.57 ± 0.57	31.07 ± 0.55	31.94 ± 1.76	28.00 ± 0.50*	41.03 ± 2.16	40.14 ± 1.41	39.53 ± 1.19	38.10 ± 1.31	37.37 ± 0.98*
BWG (%)	5.60	4.99	8.80	7.58	8.00	5.27	2.78	8.99	8.88	8.27	6.83	4.72
FI (g/day)	9.21 ± 1.03	8.11 ± 1.27	6.98 ± 0.81	6.81 ± 0.48	6.91 ± 0.63	6.53 ± 0.91	5.80 ± 1.17*	8.41 ± 0.93	7.94 ± 0.44	7.19 ± 0.59	6.67 ± 0.73	6.71 ± 0.27*
WI (mL/day)	10.13 ± 1.35	10.24 ± 1.09	10.76 ± 1.25	10.58 ± 0.78	11.02 ± 0.85	9.54 ± 1.20	7.97 ± 1.08*	12.44 ± 1.64	11.34 ± 0.48	10.95 ± 0.59	9.59 ± 1.91	8.65 ± 0.93*

Values expressed as mean ± SD

*BWG* body weight gain, *FI* food intake, *WI* water intake

*Significantly different from the control group, *p* < 0.05

**Table 2:** Relative organ weight (g/100 g of body weight) of mice treated orally with total alkaloid from *M. henryi*

Parameters (g/100 g)	Acute toxicity (mg/kg)		Sub-acute toxicity (mg/kg)									
	Female		Female					Male				
	0	2000	Control	125	250	500	1000	Control	125	250	500	1000
Heart	0.46 ± 0.03	0.47 ± 0.02	0.56 ± 0.03	0.55 ± 0.03	0.58 ± 0.04	0.57 ± 0.05	0.54 ± 0.02	0.54 ± 0.04	0.53 ± 0.03	0.54 ± 0.06	0.55 ± 0.03	0.52 ± 0.07
Liver	5.17 ± 0.12	5.32 ± 0.24	5.25 ± 0.18	5.19 ± 0.17	5.30 ± 0.36	5.54 ± 0.42	6.04 ± 0.10*	4.71 ± 0.06	4.72 ± 0.17	5.09 ± 0.41	5.11 ± 0.45	5.62 ± 0.22*
Spleen	0.32 ± 0.02	0.29 ± 0.01	0.41 ± 0.03	0.41 ± 0.03	0.43 ± 0.04	0.41 ± 0.03	0.39 ± 0.02	0.35 ± 0.02	0.36 ± 0.02	0.36 ± 0.01	0.32 ± 0.03	0.36 ± 0.01
Lung	0.61 ± 0.01	0.65 ± 0.01	0.64 ± 0.02	0.62 ± 0.03	0.69 ± 0.04	0.63 ± 0.03	0.64 ± 0.02	0.66 ± 0.01	0.67 ± 0.02	0.62 ± 0.03	0.62 ± 0.02	0.68 ± 0.04
Kidney	1.18 ± 0.06	1.28 ± 0.03	1.30 ± 0.04	1.35 ± 0.04	1.31 ± 0.06	1.32 ± 0.09	1.39 ± 0.05	1.55 ± 0.04	1.53 ± 0.03	1.52 ± 0.12	1.55 ± 0.11	1.64 ± 0.12
Ovary/Testis	0.06 ± 0.01	0.06 ± 0.01	0.14 ± 0.02	0.11 ± 0.02	0.10 ± 0.01	0.10 ± 0.01	0.10 ± 0.01	0.76 ± 0.02	0.78 ± 0.03	0.81 ± 0.04	0.79 ± 0.03	0.78 ± 0.05

Results are expressed as mean ± SD (male/female n = 5)

*Significantly different from the control group, *p* < 0.05

### Sub-Acute Toxicity Study

#### Body Weight, Food and Water Intake

During the 28 days of MHTA treatment, no treatment related toxicity signs or other behavioral and physiological changes were found in both sexes of control and treated animals at MHTA doses of 125, 250 and 500 mg/kg. However, irregular hair in the mice treated with the highest dose was observed at 2nd week. As shown in Table 1, the body weight of 125 and 250 mg/kg MHTA treated animals increased continuously, as did the control group. However, in the 500 and 1000 mg/kg groups, the weight gains of animals decreased significantly. At the end of experiment, the body weight, food intake and water consumption of animals in the groups of 1000 mg/kg were significant lower than those in the control group (*p* < 0.05), whereas a similar phenomenon was observed at 500 mg/kg group but was not significant (*p* > 0.05) (Table 1).

#### Organ Coefficient

The organs of all animals were collected and weighted after the experiment, and the relative organ coefficients were calculated as shown in Table 2. No significant differences occurred in organ coefficients (heart, spleen, lung, kidney, testis and ovary) between treated and control groups. However, the relative liver coefficient in both males and females treated with 1000 mg/kg MHTA markedly increased compared to that in mice of the control group (*p* < 0.05).

#### Hematological Analysis

The hematological parameters in animals are shown in Table 3. In both female and male mice, the WBC and LYM levels significantly increased in the groups of 500 and 1000 mg/kg MHTA in comparison with the control ones (*p* < 0.05). Besides, the LYM% and MON in females treated with 500 mg/kg MHTA as well as males and females that received 1000 mg/kg MHTA were obviously increased (*p* < 0.05) than those in mice of the control group, The GRA% and RBC significantly decreased in mice of 1000 mg/kg (both sexes) and RBC also significantly decreased in females of 500 mg/kg (*p* < 0.05). The MCV levels in 1000 mg/kg MHTA significantly increased than the control group. PLT levels significantly increased in females at dose of 500 mg/kg and all animals at dose of 1000 mg/kg, while PCT% significantly increased in the 1000 mg/kg MHTA group (*p* < 0.05). In addition, no significant changes were observed in other biochemical parameters between the control and MHTA treated mice.

**Table 3:** Hematological analysis results of mice treated orally with the total alkaloid from *M.henryi* for 28 day

Parameters	Female					Male				
	Control	125 mg/kg	250 mg/kg	500 mg/kg	1000 mg/kg	Control	125 mg/kg	250 mg/kg	500 mg/kg	1000 mg/kg
WBC 10^9^/L	6.73 ± 0.48	6.74 ± 0.40	7.31 ± 0.30	8.21 ± 0.30*	13.30 ± 0.15*	6.68 ± 0.39	6.10 ± 0.60	7.60 ± 0.55	8.40 ± 0.55*	9.87 ± 0.40*
LYM 10^9^/L	6.69 ± 0.31	6.77 ± 0.56	6.93 ± 0.42	7.53 ± 0.20*	9.53 ± 0.11*	6.94 ± 0.42	7.68 ± 0.14	7.40 ± 0.83	8.34 ± 0.65*	10.10 ± 0.50*
LYM %	67.56 ± 2.24	69.30 ± 1.70	69.90 ± 3.20	71.40 ± 1.53	79.60 ± 2.85*	69.88 ± 5.70	67.60 ± 6.05	69.50 ± 4.00	70.7 ± 3.87	77.70 ± 1.35*
MON 10^9^/L	0.66 ± 0.23	0.67 ± 0.21	0.75 ± 0.11	1.03 ± 0.10*	1.18 ± 0.15*	0.73 ± 0.24	0.73 ± 0.16	0.74 ± 0.03	0.82 ± 0.08	1.22 ± 0.12*
GRA %	29.57 ± 0.81	30.90 ± 2.90	30.00 ± 2.08	27.90 ± 1.56	26.20 ± 1.06*	25.03 ± 1.32	24.20 ± 2.10	24.20 ± 1.15	26.60 ± 2.21	29.10 ± 26*
RBC 10^12^/L	11.07 ± 0.11	12.54 ± 0.08	10.01 ± 0.52	8.59 ± 0.24*	7.77 ± 0.36*	9.48 ± 0.31	10.05 ± 0.36	9.25 ± 0.66	8.85 ± 0.36	6.94 ± 0.28*
HGB g/L	137.87 ± 9.63	139.70 ± 10.15	133.00 ± 3.85	138.09 ± 12.48	124.20 ± 11.16	135.46 ± 6.38	137.80 ± 9.25	138.40 ± 4.05	130.95 ± 8.52	121.80 ± 3.75
MCH Pg	11.13 ± 0.69	10.90 ± 0.50	11.30 ± 0.85	11.60 ± 0.75	12.90 ± 0.68	12.73 ± 1.23	13.10 ± 1.45	12.10 ± 0.15	11.30 ± 0.09	13.40 ± 0.60
MCV fL	44.35 ± 2.26	46.40 ± 3.14	45.10 ± 2.57	45.30 ± 4.02	49.81 ± 2.13*	46.19 ± 1.61	46.90 ± 2.60	46.40 ± 2.20	48.10 ± 3.25	50.60 ± 2.07*
HCT %	41.68 ± 1.32	42.80 ± 2.01	40.30 ± 0.66	39.02 ± 1.02	39.60 ± 0.96	43.35 ± 2.19	46.30 ± 3.85	41.50 ± 2.25	39.00 ± 3.02	39.59 ± 2.32
PLT 10^9^/L	443.47 ± 21.54	437.00 ± 42.00	461.00 ± 38.25	539.00 ± 36.00*	571.00 ± 22.18*	443.00 ± 31.83	426.00 ± 20.41	451.00 ± 42.00	523.00 ± 38.45	593.00 ± 36.17*
PCT %	0.54 ± 0.05	0.62 ± 0.06	0.51 ± 0.08	0.63 ± 0.06	0.67 ± 0.03*	0.81 ± 0.13	0.76 ± 0.25	0.77 ± 0.07	0.98 ± 0.06	1.22 ± 0.16*
MPV fL	5.43 ± 0.61	5.60 ± 0.25	5.90 ± 0.35	5.29 ± 0.36	5.10 ± 0.42	6.18 ± 0.49	5.60 ± 0.45	6.50 ± 0.64	6.30 ± 0.54	5.70 ± 0.30

Results are expressed as mean ± SD (male/female n = 5)

*Significantly different from the control group, *p* < 0.05

#### Serum Biochemical Parameters

In sub-acute toxicity, the serum biochemical parameters are summarized in Table 4. Both female and male mice treated with 500 and 1000 mg/kg MHTA exhibited a remarkable decrease in TP in comparison with control (*p* < 0.05). Meanwhile, ALB levels were significantly decreased in males treated with 500 and 1000 mg/kg MHTA (*p* < 0.05), and that in females were also decreased with no statistical significance. The levels of AST in 1000 mg/kg MHTA group and ALT, BUN and CRE in 500 and 1000 mg/kg MHTA groups significantly increased (*p* < 0.05) compared to the control group. Na concentrations in females treated with 1000 mg/kg MHTA statistically decreased (*p* < 0.05), while K values markedly increased (both females and males) (*p* < 0.05) in comparison with the control ones. Moreover, the parameters such asTBIL, TG, TC, GLU and Cl were found in a normal range with no significant difference to the control.

**Table 4:** Serum biochemistry results of mice treated orally with the total alkaloid from *M.henryi* for 28 days

Parameters	Female					Male				
	Control	125 mg/kg	250 mg/kg	500 mg/kg	1000 mg/kg	Control	125 mg/kg	250 mg/kg	500 mg/kg	1000 mg/kg
TP (g/L)	47.32 ± 1.19	48.06 ± 0.44	49.75 ± 1.32	43.21 ± 0.89*	40.76 ± 1.13*	49.32 ± 2.03	49.97 ± 1.20	49.26 ± 0.68	45.86 ± 0.71*	43.97 ± 0.77*
ALB (g/L)	29.11 ± 1.84	29.82 ± 0.94	30.44 ± 0.57	25.32 ± 2.04	21.32 ± 1.04*	28.79 ± 1.00	29.45 ± 0.04	29.73 ± 0.59	26.13 ± 0.38*	24.05 ± 0.25*
AST (U/L)	128.59 ± 10.36	124.75 ± 8.99	131.45 ± 14.76	147.86 ± 10.28	154.98 ± 12.13*	146.61 ± 11.01	145.17 ± 12.63	150.44 ± 13.76	159.99 ± 12.16	167.87 ± 10.96*
ALT (U/L)	42.32 ± 2.89	41.27 ± 2.81	45.10 ± 4.07	54.15 ± 4.21*	68.79 ± 7.32*	45.06 ± 1.98	43.79 ± 3.75	48.41 ± 2.48	52.68 ± 4.11*	60.97 ± 5.88*
TBIL (μmol/L)	7.93 ± 0.68	7.64 ± 0.87	8.01 ± 0.46	8.56 ± 0.71	8.97 ± 0.69	8.23 ± 0.61	8.16 ± 0.49	8.23 ± 0.67	8.89 ± 0.61	9.02 ± 0.75
TG (mmol/L)	1.07 ± 0.10	1.12 ± 0.04	1.37 ± 0.11	1.09 ± 0.08	1.22 ± 0.06	1.03 ± 0.09	1.15 ± 0.12	1.37 ± 0.05	1.32 ± 0.03	1.02 ± 0.12
TC (mmol/L)	2.13 ± 0.06	2.23 ± 0.06	2.28 ± 0.17	2.38 ± 0.36	2.21 ± 0.09	1.98 ± 0.03	2.06 ± 0.07	2.00 ± 0.11	1.90 ± 0.03	1.92 ± 0.05
CRE (mg/dL)	38.03 ± 0.91	37.21 ± 0.43	39.87 ± 1.92	44.12 ± 1.56*	49.91 ± 2.01*	45.29 ± 2.42	47.34 ± 1.13	48.14 ± 0.43	54.96 ± 1.87*	63.02 ± 0.25*
BUN(mmol/L)	6.43 ± 0.27	6.46 ± 0.12	6.74 ± 0.39	7.64 ± 0.52*	8.93 ± 0.25*	5.61 ± 0.43	5.49 ± 0.48	5.84 ± 0.31	6.65 ± 0.33*	7.93 ± 0.48*
GLU (mg/dL)	3.59 ± 0.09	3.64 ± 0.13	3.54 ± 0.25	3.56 ± 0.10	3.55 ± 0.05	4.69 ± 0.12	4.78 ± 0.09	4.84 ± 0.21	4.73 ± 0.03	4.63 ± 0.35
Na (mmol/L)	145.06 ± 11.03	147.87 ± 11.45	151.27 ± 19.48	139.64 ± 12.06	118.19 ± 12.83*	152.06 ± 8.77	148.68 ± 9.97	155.22 ± 12.74	149.87 ± 12.63	126.39 ± 13.59*
K (mmol/L)	7.71 ± 0.62	7.55 ± 0.49	7.38 ± 0.56	8.40 ± 0.43	9.57 ± 0.24*	6.83 ± 0.49	7.06 ± 0.58	6.89 ± 0.63	6.41 ± 0.29	9.12 ± 0.37*
Cl (mmol/L)	99.45 ± 6.34	101.45 ± 8.71	98.75 ± 4.96	98.01 ± 6.75	96.27 ± 7.23	100.06 ± 4.85	100.96 ± 9.01	101.87 ± 5.46	97.25 ± 6.77	94.82 ± 5.93

Results are expressed as mean ± SD (male/female n = 5)

*Significantly different from the control group, *p* < 0.05

#### Histopathological Analysis

The histopathological studies of the vital organs (kidney, liver, heart, spleen, lungs, ovary, and testis) of the control group, 125 and 1000 mg/kg MHTA groups were performed. The photomicrograph results of those vital organs were as follows: for liver parenchyma, normal morphology of hepatic lobules were observed in the control (Fig. 3A1) and lowest dose group (Fig. 3A2), while tissue necrosis, mononuclear cells infiltration, and hepatocyte apoptosis were occasionally found around the hepatic sinusoid in 1000 mg/kg MHTA treated group (Fig. 3A3). In kidney, the lowest dose group revealed no detectable abnormalities (Fig. 3B1–B2), while different degrees of hyperemia in glomerulus, inflammatory cell infiltration in renal tubular epithelial cell, and the narrowed and occlusive renal tubular were observed in mice of the highest dose (Fig. 3B3). Spleen showed normal red and white pulp as well as normal capsules in mice of the control and lowest dose group (Fig. 3C1–C2), while some focal necrosis and nuclear aggregations were observed in mice of the highest dose (Fig. 3C3). In heart, myocardium cell showed cross-over between muscle fibers in both the lowest dose and control group (Fig. 3D1–D2), while those cells exhibited nuclear aggregations and hypertrophy, and even myocardium cell apoptosis in the highest group (Fig. 3D3). In lung, all the experimental groups showed the same pattern of several alveolar air spaces separated by relatively thin alveolar septa (Fig. 3E1–E3), and no alteration was detected. Gross anatomical examinations on the ovary and testis showed no pathological lesions.

### Structural Characterization of Compounds

**Fig. 4:**
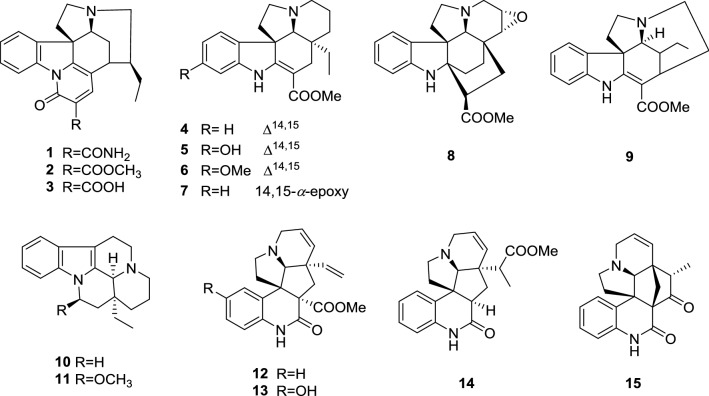
Chemical structures of compounds **1**–**15**

In order to provide the information of chemical investigations, a phytochemistry investigation of MHTA were performed. A total of 15 TIAs were isolated by a series of column chromatographic separations and purification via MPLC, Pre-HPLC, and sephadex LH-20 (Fig. 4). Their structures were determined by the comprehensive analysis of ^1^H NMR, ^13^C NMR and HRESIMS spectroscopic data (^13^C NMR data in Table 5, 1H NMRand HRESIMS data were not shown). When compared with the reported data in the literatures, the isolated pure compounds were elucidated as melodinines A-C (**1–3**) [19], tabersonine (**4**) [20], 11-hydroxytabersonine (**5**) [21], 11-methoxytabersonine (**6**) [22], lochnericine (**7**) [22], kopsinine (**8**) [23], tubotaiwine (**9**) [24], ( −)-eburnamenine (**10**) [25], *O*-methylepivincanol (**11**) [25], scandine (**12**) [26], 10-hydroxyscandine (**13**) [27], melaxilline A (**14**) [28], meloscandonine (**15**) [29].

**Table 5:** C NMR spectroscopic data of compounds **1**–**15** (δ in ppm)

NO^b^	1	2	3	4	5	6	7	8	9	10	11	12	13	14	15
2	161.2	158.7	162.0	166.8	167.6	167.2	167.7	67.0	170.5	130.2	131.3	169.2	167.3	172.2	168.9
3	62.2	60.0	62.1	50.6	50.9	50.5	50.1	48.0	45.1	45.4	45.6	47.7	47.8	44.7	47.1
5	54.5	53.6	54.3	50.9	50.5	50.8	50.6	49.3	53.8	52.1	52.3	53.3	53.2	52.9	55.0
6	44.9	46.2	44.6	44.5	44.4	44.5	44.7	36.2	43.8	16.6	17.6	39.9	41.0	39.6	40.3
7	55.6	56.6	55.6	55.1	54.5	54.4	54.8	55.9	55.0	107.1	106.2	57.7	57.6	58.1	56.8
8	140.1	139.4	139.6	138.0	129.8	130.4	137.5	139.1	137.0	128.3	130.0	128.9	127.9	126.2	130.3
9	120.1	120.3	120.5	121.4	122.1	121.7	121.4	121.2	119.6	118.5	118.9	127.0	114.3	127.8	123.6
10	126.9	126.7	128.0	120.6	107.2	105.0	120.7	119.3	121.0	119.8	121.0	123.7	153.0	124.3	123.9
11	128.1	128.6	128.6	127.6	156.4	159.9	127.7	126.9	127.2	121.5	122.2	127.5	115.8	127.7	127.9
12	117.4	118.2	117.9	109.3	97.7	96.7	109.4	111.1	109.7	108.5	111.9	115.5	113.9	115.8	116.2
13	140.7	140.8	139.7	143.2 s	144.2 s	144.3 s	142.9	149.1	143.6	133.5	137.2	134.2	130.0	135.0	136.6
14	31.4	30.9	30.9	124.8	124.7	124.8	53.9	53.6	28.3	20.8	21.6	122.9	123.4	129.1	125.8
15	36.3	33.8	36.1	133.1	133.0	133.0	57.2	58.0	30.8	27.5	26.6	131.4	131.2	132.2	128.1
16	115.7	118.7	117.3	92.1	92.1	92.2	90.6	43.3	95.6	119.7	84.1	63.7	63.7	48.6	67.8
17	145.4	143.9	146.4	26.9	28.3	26.8	23.2	26.8	11.5	116.7	35.7	44.1	44.3	41.9	38.4
18	11.5	12.8	11.4	7.49	7.5	7.4	7.2	33.8	23.8	9.0	7.9	114.6	113.8	12.8	8.5
19	26.5	27.6	26.3	28.4	26.9	28.3	24.3	27.6	41.1	31.1	29.8	142.2	142.4	47.6	52.7
20	38.7	122.8	38.3	41.3	41.3	41.3	41.0	35.9	65.4	37.3	35.9	46.6	47.2	45.8	45.5
21	51.5	129.9	51.2	70.0	70.2	70.1	67.5	61.6	72.8	55.8	60.4	83.6	84.3	75.8	61.6
22	161.6	159.5	163.3	–	–	–	–	–	–	–	–	–	–	–	–
23	120.2	119.4	117.1	–	–	–	–	–	–	–	–	–	–	–	–
*CO*_*2*_*NH*_*2*_	165.7	–	–	–	–	–	–	–	–	–	–	–	–	–	–
*C*O_2_Me	–	166.1	–	169.0	169.4	169.0	168.8	174.1	168.8	–	–	170.4	170.6	174.6	–
CO_2_*Me*	–	52.3	–	51.0	51.3	51.0	51.1	52.0	51.1	–	–	52.6	51.6	51.4	–
*CO*_*2*_*H*	–	–	165.5	–	–	–	–	–	–	–	–	–	–	–	–
*CO*	–	–	–	–	–	–	–	–	–	–	–	–	–	–	209.5
*OMe*	–	–	–	–	–	55.4	–	–	–	–	55.8	-	-	-	-

Data were measured at 150 MHz for ^13^C NMR.

^a^All compounds were measured in CDCl_3_

## Discussion

People also depend on traditional herbal medicine to treat diseases in developing countries [30]. Plants are invaluable sources of pharmaceutical agents [31], especially alkaloids [32]. *M. henryi* has been proved to be a good source of TIAs and has been used as a folk medicine for the treatment of some inflammatory diseases. Previous investigations on *M. henryi* are mainly focused on the isolation of TIAs with novel structures, evaluation of their cytotoxic activities on the human cancer cell lines. However, the researches on the anti-inflammatory potentials of the bioactive extract, and their phytochemicals have not been exploited. In this paper, *M. henryi* was extracted by methanol to obtain the crude extract (CE), and then processed to yield the non-alkaloidal extract (MHNA) and total alkaloidal extract (MHTA). We firstly reported the anti-inflammatory and immunosuppressive activities of *M. henryi*. The result showed that MHTA had the strongest anti-inflammatory and immunosuppressive activity than CE and MHNA.

In order to provide the information of MHTA for the treatment of disease, the preclinical safety assessment is necessary to performed. The acute toxicity test was initially performed in mice of females due to the higher sensitivity than males, as it provides data to guide the other toxicity tests [32]. There were no significant changes in clinical signs, body weight gain, and food and water intakes in any female mice treated with 2000 mg/kg MHTA. The organs did not show any abnormality in their gross pathology examination. Therefore, the LD_50_ of the MHTA can be considered greater than 2000 mg/kg, which was regarded as practically low toxic.

In the sub-acute toxicity experiments, the decreases of food consumption and water intake were observed in the dose of 1000 mg/kg during the experiment period (*p* < 0.05). The body weight of all animals in the 1000 mg/kg group was significantly lower compared to the control (*p* < 0.05), meaning that high dose of MHTA may be toxic to mice. The organ coefficient is a crucial detection index in the evaluation of drug safety, and provides a quantitative reference for drug evaluation. When the relative organ weights significantly changes, some underlying disease or damage (congestion, edema, or hypertrophy) may happen [33]. In this study, the relative weights of the liver in mice of 1000 mg/kg group were significantly higher than that in mice of the control group (*p* < 0.05), suggesting that doses of 1000 mg/kg MHTA might induce liver damage. The liver played a key role in the biotransformation, detoxification, and synthesis of serum proteins [34]. To further clarify whether MHTA has toxic effects on the liver or other organs, the hematological, serum biochemical and histopathological parameters were investigated.

The hematological parameters could provide direct evidence for toxicity assessment in humans and animals [35]. The significant increases of WBC (*p* < 0.05) in 500 and 1000 mg/kg groups were most likely induced by the body inflammation, which could be partly confirmed by the significant increases of LYM and MON. RBC and HGB levels were also significantly decreased at high doses of MHTA, and was in agreement with the previously reported damages in spleen [36]. In addition, PLT was significantly increased in the high dose group (500 and 1000 mg/kg) (p < 0.05), while GRA was slightly decreased, indicating that the reduction of granulocytes led to the suppression of immune function [33]. Pervious study had showed that the exposure to pyrrolizidine alkaloid resulted to hematopoietic disfunction or erythrocyte destruction [37]. Therefore, the MHTA-induces spleen damage mechanism may be similar to that of pyrrolizidine alkaloid. Meanwhile, the activity of adrenocortical hormone may initiate negative feedback regulation, thereby inhibit the productions of red blood cells and hemoglobin [38]. Moreover, a significant increase of PCT in 1000 mg/kg group (both male and female) may be caused by the increase of PLT, which is consistent with the reported results [39]. Daily oral administration of MHTA at high concentration for 28 days may affect the glucocorticoid hyperactivity, and lead to liver damage.

In the analysis of biochemical indicators, the TP, composed of ALB and GLB, in the groups of 500 and 1000 mg/kg decreased significantly (*p* < 0.05), suggesting that the ability of protein synthesis may be reduced. ALB shows a downward trend, suggesting the rise of GLB in the 500 and 1000 mg/kg groups. The increase of GLB is a sign of activation of the immune system, indicating the occurrence of inflammation in the body [40], which was consistent with the predicted increases of WBC and LYM levels.

The liver is the main organ responsible for natural products metabolism and increased indicative activities of serum enzymes AST and ALT are considered sensitive markers of hepatocellular damage [41]. Higher levels of AST and ALT were observed in higher dose group compared to the control (*p* < 0.05), indicating that high doses of MHTA might damage the liver. The observed increase in the relative liver organ weight and reduction of RBC confirmed this assumption, which was consistent with the reported result [42]. The kidney is another sensitive organ exposed to toxic compounds. CRE and BUN are known as effective indicators for assessing kidney injury [43]. The levels of CRE and BUN in the 500 and 1000 mg/kg MHTA groups significantly increased than those in the control group (*p* < 0.05). Meanwhile, the content of sodium ions significantly decreased, while the content of potassium ions significantly increased in the highest dose group, indicating that the kidney was damaged and glomerular filtration function injury resulted in the impairment of ion balance in the blood [40]. This finding was further confirmed by histopathological observations of the kidney tissue in this study.

Biochemical data of high concentrations of MHTA revealed side effects to vital organs in mice, the histopathological studies provide a supportive evidence for hematological and biochemical analysis. The histopathology changes mainly occurred in the liver, kidney, spleen, and heart at high doses of MHTA (1000 mg/kg). Meanwhile, the severity of the dose is related to increased treatment duration, suggesting accumulative toxic effect of the alkaloids.

Furthermore, there is an urgent need for phytochemical investigation on bioactive ingredients of this plant to understand the chemical components of MHTA. Phytochemical research showed that about 66 alkaloids of different structures have been isolated from *M. henryi* so far [8, 12–14, 19]. In this study, 15 monoterpenoid indole alkaloids were isolated and identified. According to the structural characteristics, the compounds were classified as strychnos-type alkaloids (**1**–**3**), aspidosperma-type alkaloids (**4**–**8**), condylocarpan-type alkaloid (**9**), eburna-type alkaloids (**10**–**11**), and quinoline-type alkaloids (**12**–**15**).

Previous reports on the cytotoxic activities of the compounds showed that aspidosperma-type alkaloids **4**, **5**, **6** and **8** had significant cytotoxic activities on human cancer cell lines, while other types of compounds have no obvious cytotoxic activity [13, 19]. Therefore, the chemical constituents and contents of aspidosperma-type alkaloids may be related to the toxicity of MHTA. Besides, some quinoline and indole alkaloids isolated from *Melodinus* plants, such as 15-dihydroscandine, 15β-hydroxy-14,15-dihydroscandine, melaxillines A and B, melodinine T, scandine, 11-hydroxytabersonine, and *O*-methyl-Δ^14^-vincanol-3-one exhibited significant anti-inflammatory activity [17, 28, 44, 45]. Among the isolates in this study, tabersonine (**4**) could attenuate LPS-induced acute lung injury and inhibited LPS-mediated macrophage activation in vitro [46], 11-hydroxytabersonine (**5**) showed inhibitory effect on NO production [17], tubotaiwine (**9**) showed good inhibitions on COX-1 and COX-2 in anti-inflammatory assay in vitro [47], and melaxilline A (**14**), scandine (**12**), and meloscandonine (**15**) significantly inhibited in release of β-glucuronidase [28], suggesting these compounds may be responsible for the anti-inflammatory effect of MHTA.

As we all known, the combined effects (such as synergistic effect, additive effect, antagonistic effect) in the mixture of compounds are exist in bioactivity evaluation [48], and the compounds might be absorbed, distributed, metabolized and excreted in the animal body. Whether there are some combined effects of the molecules for the toxicity and the bioactive potential still needs to be further studied.

## Conclusions

In the present study, we firstly performed the anti-inflammatory and immunosuppressive activities of *M. henryi* to fill its gap in the traditional applications. MHTA showed the strongest anti-inflammatory and immunosuppressive activities than CE and MHNA. In order to obtain the safety information of MHTA in the treatment of inflammatory diseases, the acute and sub-acute toxicity evaluations were conducted in animal models. It did not display any abnormal behaviors and mortality when administered in a single dose of 2000 mg/kg. However, the repeated administration of sub-acute toxicity experiment, MHTA (greater than 500 mg/kg) might selectively damage the organ tissues, such as kidney, heart, liver, and spleen, suggesting caution is required for the use. In addition, 15 pure TIAs were also isolated and further performed as standards for the control of the MHTA quality. Therefore, the present work provides deeper knowledge about the species *M. henryi*, expands the applications of the MHTA on the inflammatory diseases under a safe dose as a quality controllable drug candidate.

## Materials and Methods

### Samples

The aerial part of *M. henryi* (10 kg) was collected from Jinghong of China (GPS: 21°32′N/100°28′E) in October 2017, and identified by Dr. Y.P. Liu, Kumming Institute of Botany, CAS. A voucher specimen (No. Cheng20171008-03) was stored in the cool and dry environment of the faculty of agriculture and food, Kunming University of science and technology.

### Extraction and Preparation of Sample

The sample was cut into pieces, powdered and extracted with 90% aqueous CH_3_OH for three times (48 h at a time). The extract solvent was collected and then concentrated under vacuum to obtain a crude extract (CE). The residue was dissolved in acidic aqueous solvent including 0.3% hydrochloric acid (v/v) and partitioned with EtOAc (1:1, v/v) for three times. The acidic aqueous solution was adjusted with 5% ammonia to have a pH value about 9–10. The alkaline aqueous solution was then partitioned with EtOAc (1:1, v/v) for three times. After collection and concentration of EtOAc solvent, the total alkaloidal extract was obtained (MHTA). The remaining part was evaporated under reduced pressure at 50 °C to obtain the non-alkaloid extract (MHNA).

### Anti-Inflammatory Assay

RAW264.7 macrophages were purchased from Kunming cell bank, CAS, and were cultured in DMEM with 10% fetal bovine serum (FBS) and 1% antibiotics (100 U/mL penicillin and 100 μg/mL streptomycin) under a humidified atmosphere containing 5% CO_2_ at 37 °C.

Cytotoxicity of CE, MHTA and MHNA on RAW264.7 macrophage cells was performed by MTT assay [49]. Then the anti-inflammatory effect was assessed under non-toxic concentrations. In brief, after 2 h treatment with the test sample, LPS was added into the RAW264.7 cells with a concentration of 1.0 μg/mL, and the cells were cultured for 22 h. Then the content of NO was determined by the instruction of nitric oxide (NO) assay kit (Nitrate reductase method). And the TNF-α and IL-6 levels in the supernatants were measured by ELISA kits (MultiSciences (Lianke) Biotech, Hangzhou, China).

### Splenocyte Proliferation Assay

A previously reported CCK-8 method was used to carry out the immunosuppressive activity [50]. In brief, splencoytes isolated from male BALb/c mice were seeded into 96-well flat-bottom microtiter plates at a density of 1 × 10^6^ cell/mL. Then cells were exposed to the test samples in the presence of concanavalin A (Con A) and lipopolysaccharides (LPS), respectively, using the Con A-/LPS-treated splenocytes as the experimental control, dexamethasone (DXM) as positive control. After incubation for 44 h, 10 μL of CCK-8 was added and incubated for another 4 h and the absorbance was measured at 450 nm by using a microplate reader (SpectraMax M5, Molecular Device, USA). Finally, the inhibitory effects of different sample were analyzed.

### Toxicological Evaluations

#### Animals

Sixty mice (45 females and 25 males) weighing between 18 and 22 g provided by Kunming Medical University (SYXK, 2011–0004) were used. The animals were acclimated in a specificpathogen free (SPF) laboratory (12 h/12 h of dark–light cycle, temperature of 24 ± 2ºC, relative humidity of 40–70%), and fed a basic animal feed (5% fiber, 5% fat, 20% protein, 60% carbohydrate) for 7 days. The toxicological evaluations were conducted according to the recommendations from the National Institutes of Health Guide for Laboratory Animal Care and Use.

#### Acute Toxicity Evaluation

Twenty female mice were used to evaluate the acute toxicity study due to the OECD423 guideline [34]. The animalswere randomly grouped into control, 300, 1000, and 2000 mg/kg experimental groups (n = 5). The MHTA was dissolved in distilled water, and then the animal was given orally in MHTA groups, while the control group was given the medium. After administration, the general behavioral changes (i.e. altered locomotion, breathing difficulty, tremors, lethargy and convulsion) and mortality were observed at 0.5 h, 2 h, 4 h, 6 h, 10 h, and 24 h for the first day, and then recorded daily for 14 days [51]. Body weight, food and water intakes were assessedat the 4th, 7th, 11th and 14th day, and then weekly changes were calculated. The median lethal dose (LD_50_) was assessed under the principle of “Acute Toxic Class Method”.

#### Sub-Acute Toxicity Evaluation

The sub-acute toxicity experiment was performed in mice (25 females and 25 males) according to the OECD guideline [52]. The mice were randomly divide into 5 groups including the control group and four MHTA intervention groups (125, 250, 500 and 1000 mg/kg, respectively) with 5 males and 5 females per group. Each animal was administered by oral gavage daily for 28 days. The mortality and abnormal behaviors were recorded every day. Body weights, food and water intakes were assessed once a week. The mice were given a certain amount of food (water), and the amount of remaining food (water) was measured at the same time in the next day. The food (water) consumption was calculated by the difference. After 4 week of MHTA administration, all animals were anaesthetized with chloral hydrate and sacrificed by cervical dislocation. Blood was drawn in tubes containing EDTA for biochemistry and hematology assessments. The body organs (lung, spleen, heart, kidney, liver, ovary and testis) were harvested for organ index calculations, and further histo-pathological analyses.

#### Hematological Analysis

The hematological parameters, including percentage of lymphocytes (LYM%), lymphocyte (LYM), red blood cell (RBC), red blood cell volume (HCT), white blood cell (WBC), monocytes (MON), mean corpuscular hemoglobin (MCH), percentage of granulocytes (GRA%), mean platelet volume (MPV), mean corpuscular volume (MCV), platelet (PLT), hemoglobin (HGB), and platelet pressure (PCT) were detected by an automatic blood cell analyzer HF-3800 Plus (Hanfang Ltd., Jinan, China).

#### Biochemical Analysis

The blood samples were centrifuged at 4000 rpm for 10 min at 4 °C for biochemical analysis. The serum biochemical parameters, including aminotransferase (ALT), aspartate transaminase (AST), total protein (TP), albumin (ALB), triglycerides (TG), total cholesterol (TC), total bilirubin (TBIL), glucose (GLU), alanine blood urea nitrogen (BUN), creatinine (CRE), potassium (K), sodium (Na), and chloridne (Cl) were analyzed by using commercial kits (Nanjing Jiancheng Biological Product, China).

#### Histological Analysis

The tissues (lung, kidney, heart, spleen, liver, ovary, and testis) were collected, and weighed to calculate organ coefficients [40]. All tissues were fixed in 10% buffered formalin for further hematoxylin–eosin (H&E) assessment. Then, the tissue sections were detected and analyzed for cellular damage or change under microscope.

### Isolation and Purification

The crude alkaloidal extract (98.0 g) was separated on a RP-C_18_MPLC column with a gradient solvent MeOH/H_2_O (from 20:80 to 100:0) to afford six fractions (A-F). Fraction A (2.6 g) was isolated on a silica gel column (30:1) with petroleum ether-acetone (v/v, 10:1, 5:1 and 3:1) and then purified by Sephadex LH-20 (MeOH) column and a preparative HPLC (CH_3_CN/H_2_O: 50:50) to yield compound **4** (9.2 mg) and **5** (28.7 mg) **7** (4 mg), **8** (13.6 mg), and **10** (28.4 mg). Fr.B (9.7 g) was chromatographed by MPLC (MeOH/H_2_O, 30:70 − 70:30) to yield three subfractions (B1–B3). Compounds **2** (33 mg) and **6** (19.6 mg) were obtained from sbufraction B-3 (203 mg) by a preparative HPLC purification (CH_3_CN/H_2_O, 40:60). Fr.C (7.7 g) was separated by MPLC (CH_3_OH/H_2_O, 30:70–70:30) and further purified by silica gel column using a petroleum ether-acetone (3:1) to give compounds **1** (15 mg), **13** (17 mg) and **11** (29 mg). Fr.D (2.6 g) was separated by MPLC with CH_3_OH/H_2_O (30:70–50:50) and further resolved by HPLC withCH_3_OH/H_2_O (50:50) to yield compounds **3** (28 mg), and **14** (3.6 mg). Fr.E (6.3 g) was separated by MPLC with CH_3_OH/H_2_O (20:80–60:40) to get compound **9** (2.9 mg) and **12** (21.2 mg). Fr.F (30 g) was separated by MPLC with CH_3_OH/H_2_O (20:80–50:50) and purified by a silica gel column (CHCl_3_: MeOH, 10:1) to afford compound **15** (8.8 mg).

### Statistical Analysis

All tests were carried out in triplicate and data were expressed as mean ± SD (standard deviation). Statistical analysis was performed with one-way analysis of variance (ANOVA). Statistical significance of differences between the groups was assessed using the Student's t-test. The *p* < 0.05 was considered significant.
